# Supplementary material for: Tumoral C2 Regulates the Tumor Microenvironment by Increasing the Ratio of M1/M2 Macrophages and Tertiary Lymphoid Structures to Improve Prognosis in Melanoma
Source: Cancers (Basel). 2024 Feb 23;16(5):908. doi: 10.3390/cancers16050908 (PMC10930905; doi:10.3390/cancers16050908)
Supplement: Supplementary file 1 [file cancers-16-00908-s001.zip › cancers-2814023-supplementary.pdf]

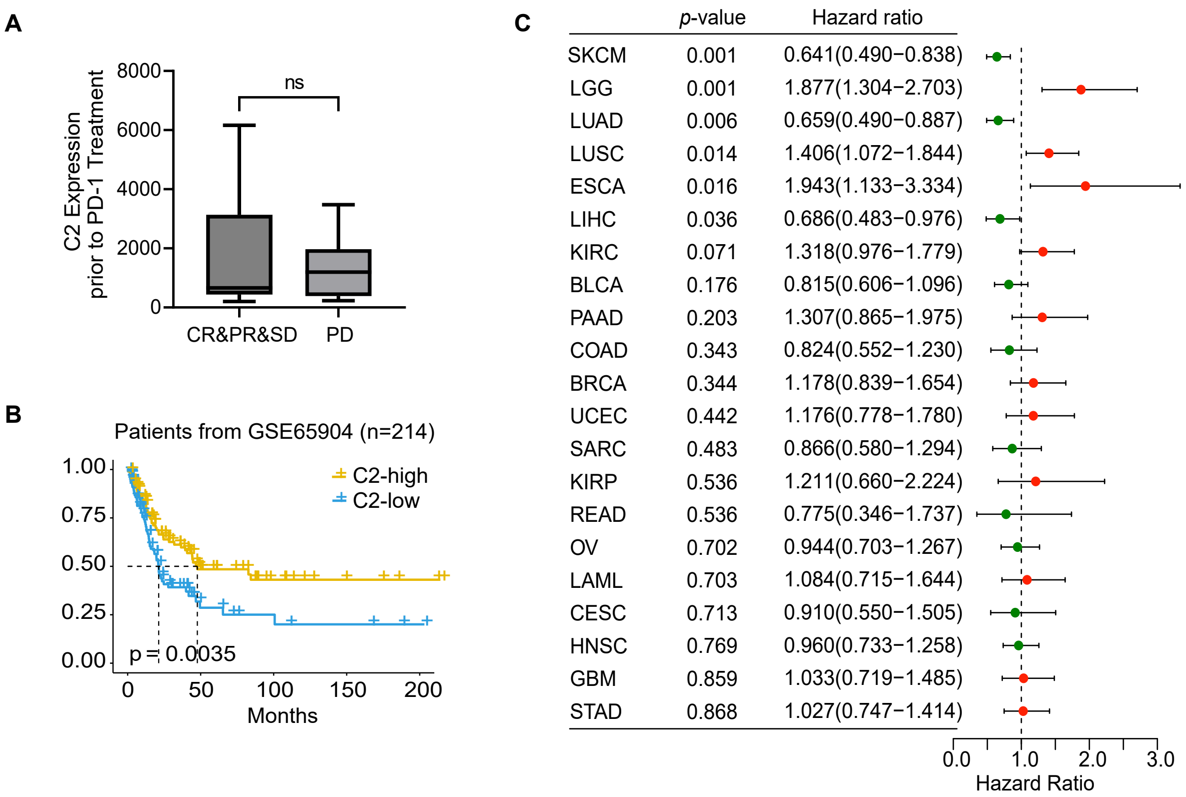


Supplementary Figure S1. Additional analysis of clinical information. (A) Comparison of baseline C2 RNA levels prior to PD-1 drug therapy. (B) Kaplan-Meier OS for different levels of C2 expression is shown based on the log-rank statistic in GSE65904. (C) Forest diagram of survival analysis in pan-cancer.

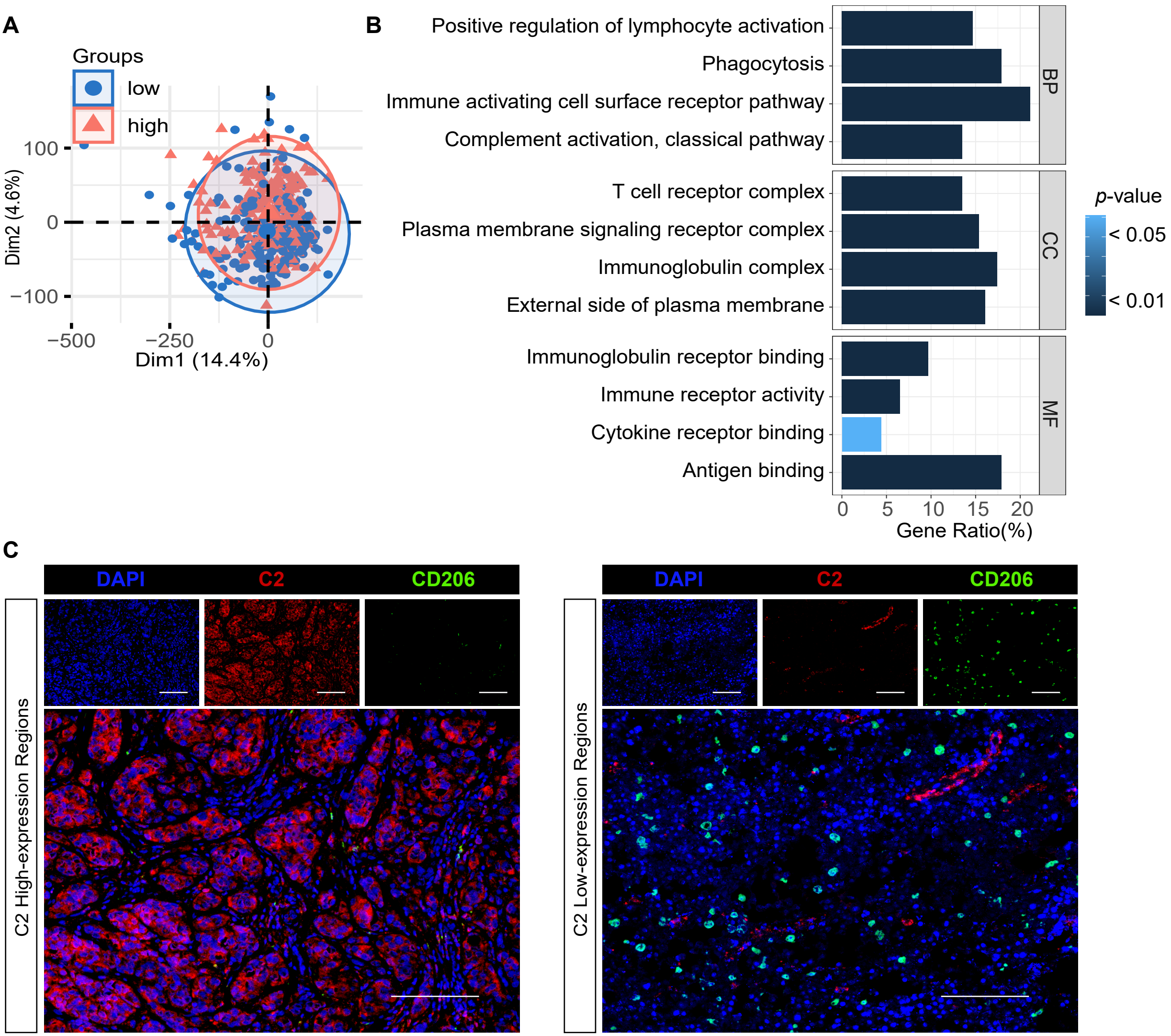


Supplementary Figure S2. Correlation of macrophages with C2 expression. (A) Principal components from the principal component analysis (PCA) based on highly differential genes in Deseq2 and edgeR methods. (B) Elevated genes with the C2 were analyzed in GO enrichment. The letters BP, CC, and MF stand for biological processes, cellular elements, and molecular functions, respectively. (C) Immunofluorescence staining demonstrated a negative correlation between C2 expression and CD206-positive cells (M2-like macrophages). Scale bar, 100 μm.

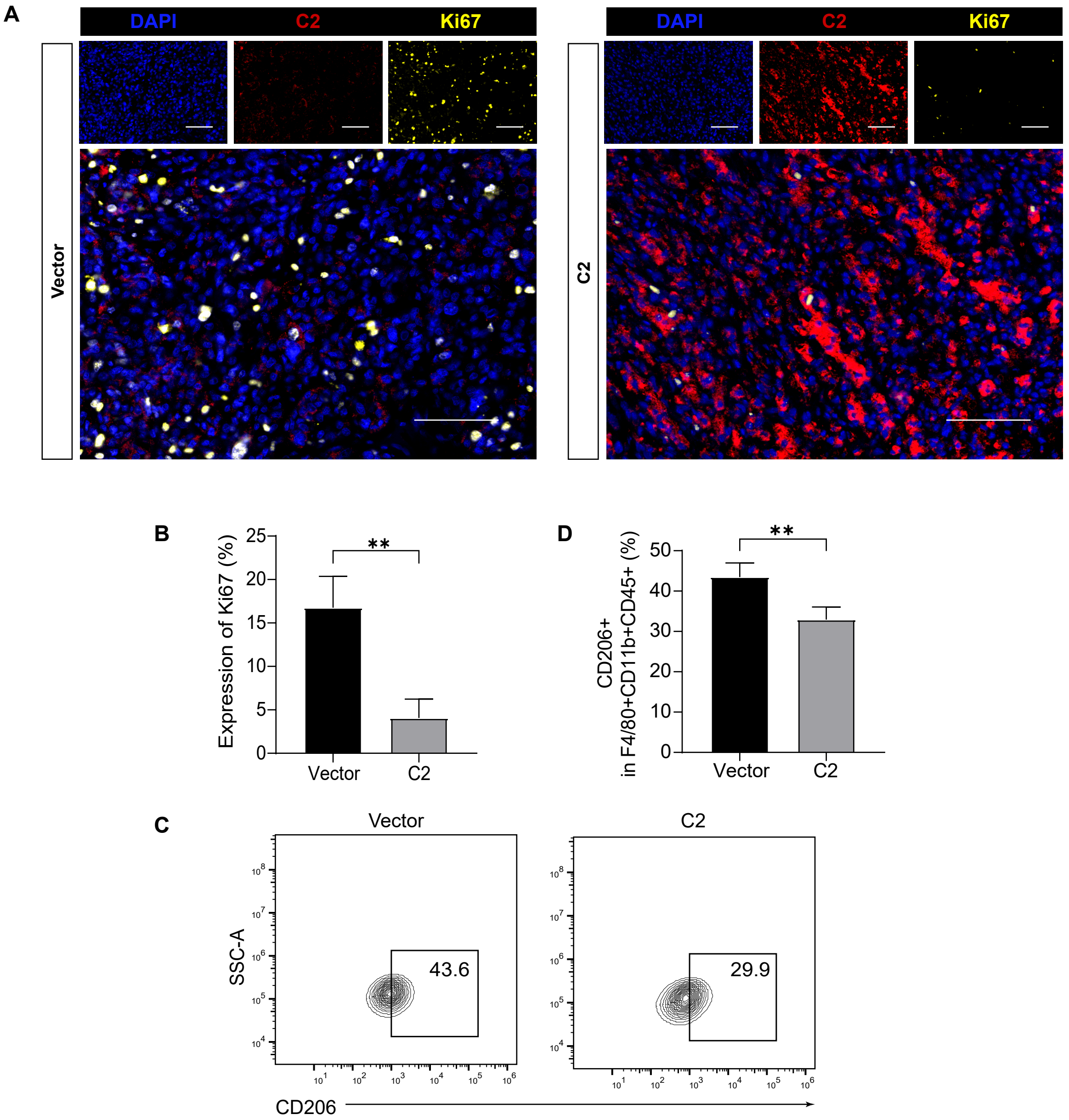


Supplementary Figure S3. Correlation between C2 expression and tumor growth retardation. (A) Distribution of Ki67-positive cells in mouse models. Scale bar, 100 μm. (B) Differences of Ki-67 positivity in murine subcutaneous model. (C-D) Flow cytometric analysis and quantification of CD206+ macrophages within F4/80+CD45+CD11b+ fraction in vector and C2 tumors. **, $p < 0.01$.

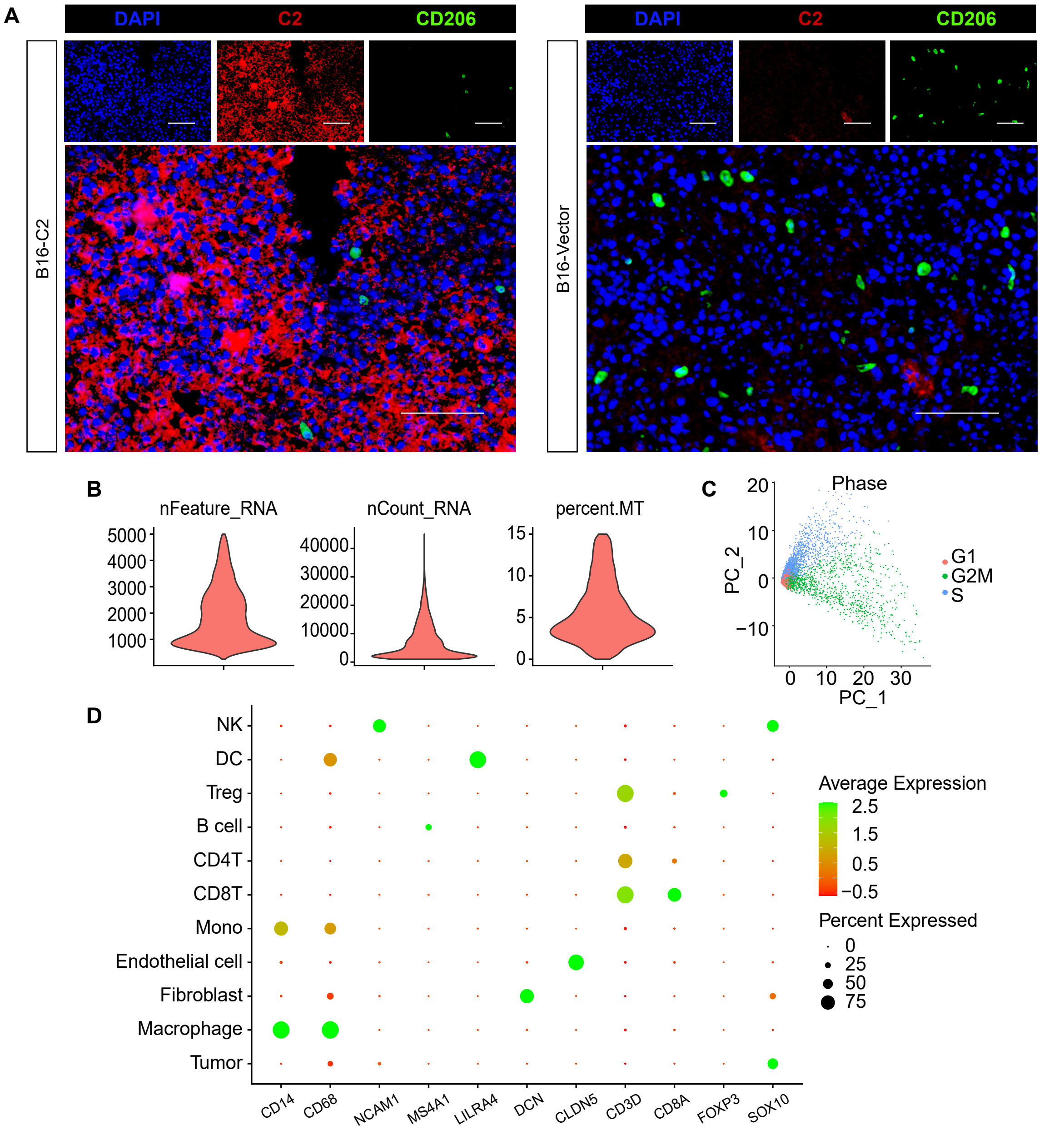


Supplementary Figure S4. Immunofluorescence in animal models and processing for single-cell sequencing analysis. (A) Distribution of CD206-positive cells in mouse models. Scale bar, 100 μm. (B) Cell screening before single-cell sequencing analysis. (C) Principal component analysis plot showing the cell cycle of the cells included. (D) Bubble heatmap depicting the expression levels of cluster-specific marker genes. The dot size indicates the fraction of expressing cells, and the dots are colored based on average expression levels.

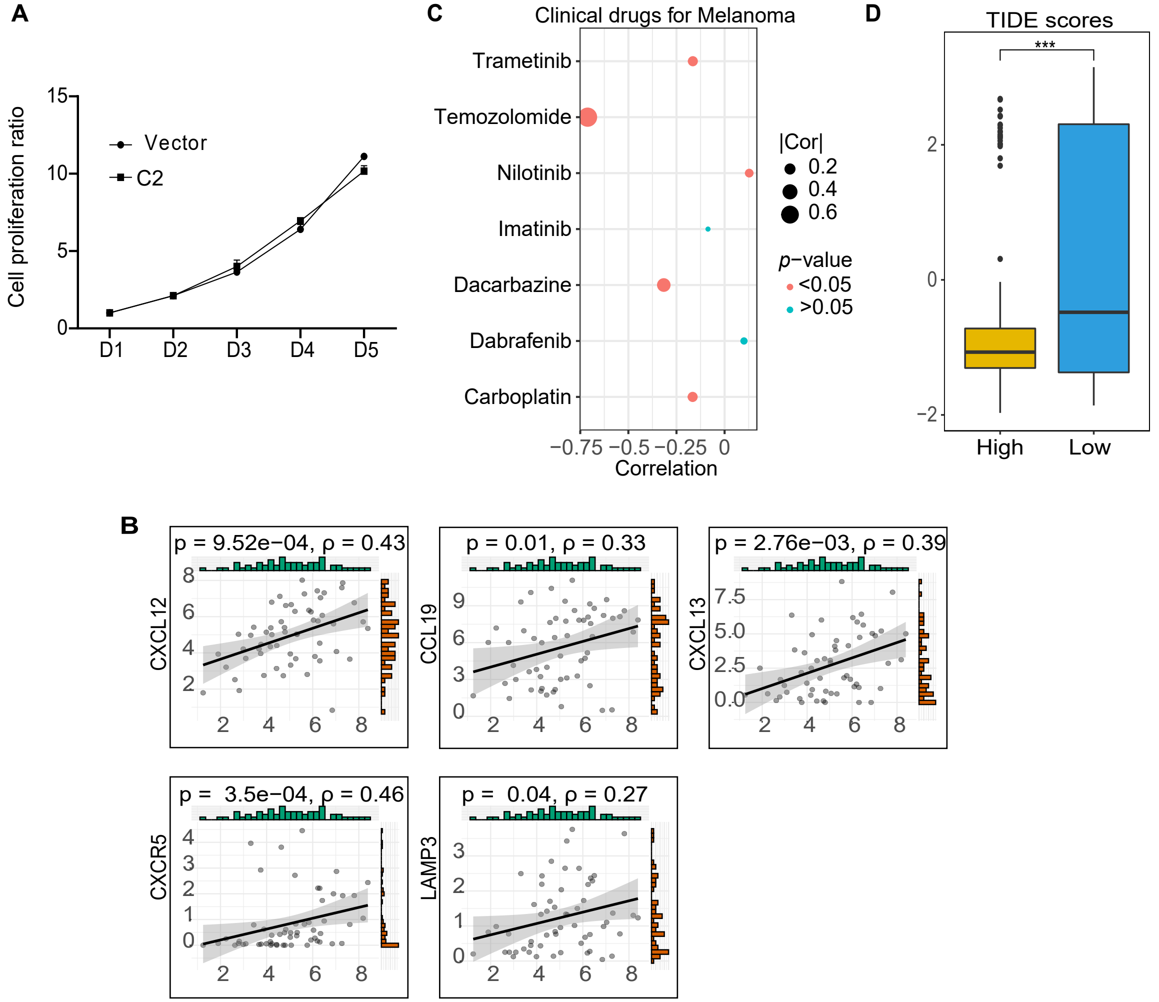


Supplementary Figure S5. C2 functional validation and drug susceptibility analysis. (A) Cell proliferation was detected by CCK8 assay in B16-C2 and B16-vector cell lines. (B) The positive association between C2 expression and marker genes for tertiary lymphoid structures verified in inner RNA-seq datasets. (C) Correlation between C2 and IC50 of clinical medicine in melanoma. (D) Predictive efficacy of anti-PD-1 and anti-CTLA-4 therapy in TIDE.

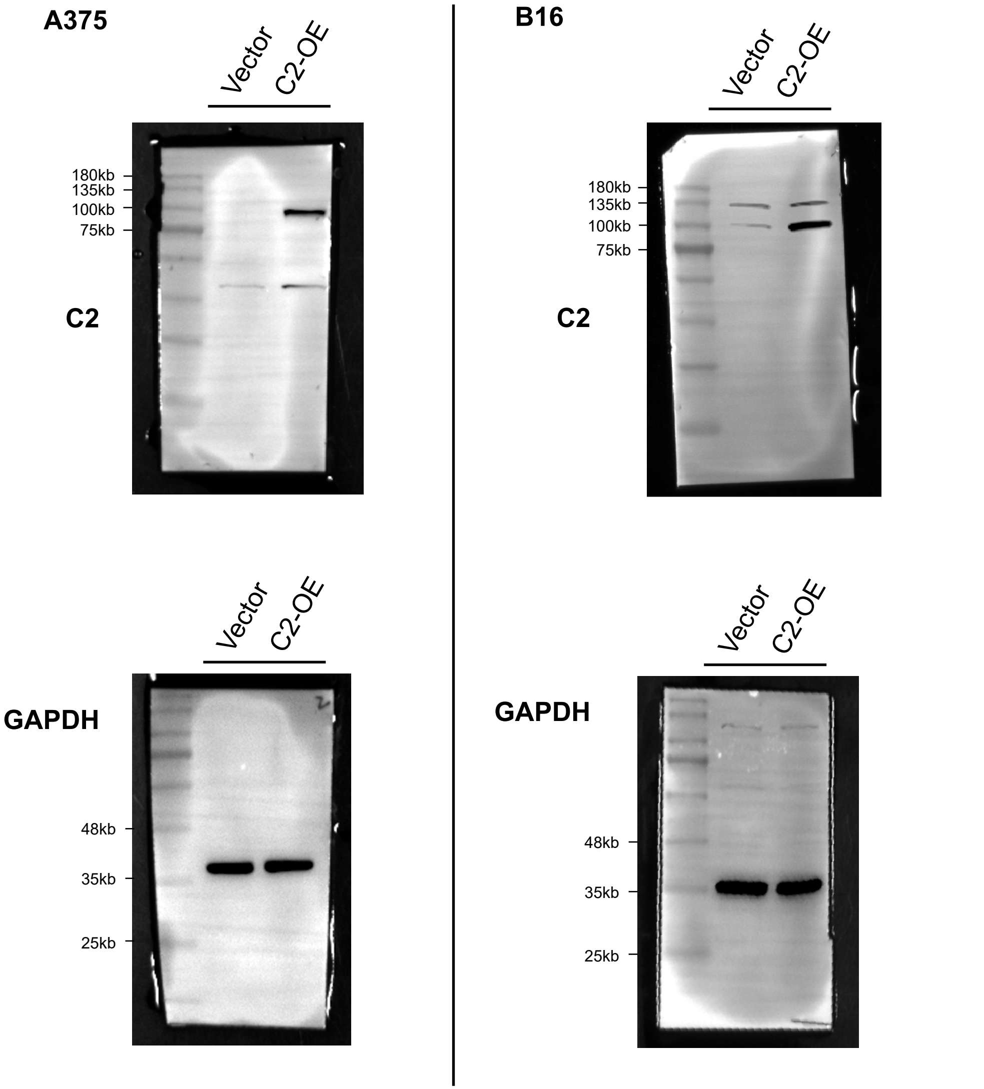


Supplementary Figure S6. Western-blot of overexpression-C2 and GAPDH in A375 and B16.

Table S1. Primers used for RT-qPCR in this study

| **Gene name** | **Forward** | **Reverse** |
|---|---|---|
| CD86 | CTGCTCATCTATACACGGTTACC | GGAAACGTCGTACAGTTCTGTG |
| TNF-α | CCTCTCTCTAATCAGCCCTCTG | GAGGACCTGGGAGTAGATGAG |
| CD206 | TCCGGGTGCTGTTCTCCTA | CCAGTCTGTTTTTGATGGCACT |
| IL-10 | TCAAGGCGCATGTGAACTCC | GATGTCAAACTCACTCATGGCT |
| C2 | TGGAAAGTCCAATATGGGTGGC | CTGGTGCAGAGCCTTTGTGT |
| IL23A | CTCAGGGACAACAGTCAGTTC | ACAGGGCTATCAGGGAGCA |
| IL12B | ACCCTGACCATCCAAGTCAAA | TTGGCCTCGCATCTTAGAAAG |
| β-Actin | CATGTACGTTGCTATCCAGGC | CTCCTTAATGTCACGCACGAT |

Table S2. TCGA cancer name and abbreviation correspondence table

| Abbreviation | Full name |
|---|---|
| BRCA | breast carcinoma |
| CESC | cervical squamous cell carcinoma |
| COAD | colon adenocarcinoma |
| DLBC | diffuse large B-cell lymphoma |
| ESCA | esophageal carcinoma |
| GBM | glioblastoma multiforme |
| HNSC | head and neck squamous cell carcinoma |
| KIRC | kidney renal clear cell carcinoma |
| KIRP | Kidney renal papillary cell carcinoma |
| LAML | acute myeloid Leukemia |
| LGG | low-grade glioma |
| LIHC | liver hepatocellular carcinoma |
| OV | ovarian serous cystadenocarcinoma |
| PAAD | pancreatic adenocarcinoma |
| PRAD | prostate adenocarcinoma |
| READ | rectum adenocarcinoma |
| SKCM | skin cutaneous melanoma |
| STAD | stomach adenocarcinoma |
| TGCT | testicular germ cell tumor |
| THCA | thyroid carcinoma |
| THYM | thymoma |
| UCEC | uterine corpus endometrial carcinoma |
| ACC | adrenocortical carcinoma |
| CHOL | cholangiocarcinoma |
| KICH | kidney chromophobe |
| LUSC | lung squamous cell carcinoma |
| LUAD | lung adenocarcinoma |

Table S3. Correlation analysis of C2 protein expression levels with CD206 in human tissues.

| | | C2 Expression | | Total | *p* |
|---|---|---|---|---|---|
| | | Low | High | | |
| CD206 | - | 1 | 8 | 9 | 0.008 |
| | + | 9 | 3 | 12 | |
| | Total | 10 | 11 | 21 | |

“-” represents <25 cells; “+” represents ⩾25 cells

Table S4. Correlation analysis of C2 protein expression levels with CD206 in murine tissues.

| | | Group | | Total | *p* |
|---|---|---|---|---|---|
| | | B16+vector | B16+C2 | | |
| CD206 | - | 0 | 4 | 4 | 0.048 |
| | + | 5 | 1 | 6 | |
| | Total | 5 | 5 | 10 | |

"-" represents <10 cells; "+" represents ⩾10 cells
